# Crystal Structure of the *Neisseria gonorrhoeae* MtrD Inner Membrane Multidrug Efflux Pump

**DOI:** 10.1371/journal.pone.0097903

**Published:** 2014-06-05

**Authors:** Jani Reddy Bolla, Chih-Chia Su, Sylvia V. Do, Abhijith Radhakrishnan, Nitin Kumar, Feng Long, Tsung-Han Chou, Jared A. Delmar, Hsiang-Ting Lei, Kanagalaghatta R. Rajashankar, William M. Shafer, Edward W. Yu

**Affiliations:** 1 Department of Chemistry, Iowa State University, Ames, Iowa, United States of America; 2 Department of Physics and Astronomy, Iowa State University, Ames, Iowa, United States of America; 3 Bioinformatics and Computational Biology Interdepartmental Graduate Program, Iowa State University, Ames, Iowa, United States of America; 4 NE-CAT and Department of Chemistry and Chemical Biology, Cornell University, Argonne National Laboratory, Argonne, Illinois, United States of America; 5 Department of Microbiology and Immunology, Emory University School of Medicine, Atlanta, Georgia, United States of America; 6 Laboratories of Microbial Pathogenesis, VA Medical Center, Decatur, Georgia, United States of America; Arizona State University, United States of America

## Abstract

*Neisseria gonorrhoeae* is an obligate human pathogen and the causative agent of the sexually-transmitted disease gonorrhea. The control of this disease has been compromised by the increasing proportion of infections due to antibiotic-resistant strains, which are growing at an alarming rate. The MtrCDE tripartite multidrug efflux pump, belonging to the hydrophobic and amphiphilic efflux resistance-nodulation-cell division (HAE-RND) family, spans both the inner and outer membranes of *N. gonorrhoeae* and confers resistance to a variety of antibiotics and toxic compounds. We here report the crystal structure of the inner membrane MtrD multidrug efflux pump, which reveals a novel structural feature that is not found in other RND efflux pumps.

## Introduction

*Neisseria gonorrhoeae* is a Gram-negative obligate human pathogen. It is the causative agent of the sexually-transmitted disease gonorrhea and rare cases of disseminated disease. Although gonorrhea is one of the oldest described diseases, it remains a significant global problem with more than 100 million cases reported annually worldwide and antibiotic resistance is a major concern [1]. The gonococcus employs a number of strategies to evade host attack. It possesses an intricate mechanism of antigenic variability through differential expression of the genome and can easily acquire new genetic material to develop resistance to antimicrobial agents [1], [2]. Gonococci utilize a number of resistance mechanisms, including antimicrobial inactivation, target modification and strategies that reduce antimicrobial concentration, such as reduced permeability of the cell envelope mediated through alteration of porin proteins and active export of multiple antimicrobial compounds from the cell by efflux pumps. Among these different mechanisms, multidrug efflux is considered to be one of the major causes of failure of drug-based treatments of infectious diseases, which appears to be increasing in prevalence [3]. These bacterial multidrug efflux pumps have enormous clinical consequences. Simultaneously rendering the cell resistant to multiple structurally-unrelated compounds, their expression results in bacterial strains resistant to most clinically-relevant antibiotics [3].

The best characterized and most clinically important of these multidrug efflux systems in *N. gonorrhoeae* is the MtrCDE tripartite efflux pump [4]–[7]. It is composed of the MtrD inner membrane transporter, belonging to the HAE-RND protein family [8]; the MtrC periplasmic protein, a member of the membrane fusion protein family; and the MtrE integral outer membrane channel protein. This system provides resistance to a broad spectrum of antimicrobial agents, including bile salts, fatty acids, dyes, antibiotics and spermicides. The Mtr multidrug efflux system is also responsible for resistance to host-derived cationic antimicrobial peptides [7], which are important mediators of the innate host defense. Given that gonococci commonly infect mucosal sites bathed in fluids containing such peptides, the Mtr system indeed underscores the pathogenesis of gonococcal disease and its contribution to virulence. In addition, it has been shown that the MtrCDE tripartite efflux pump is capable of enhancing long-term colonization of the mouse vaginal mucosal layer and that gonococci lacking this efflux pump were highly attenuated [9].

At present, only two crystal structures of HAE-RND-type efflux pumps are available. These efflux pumps are the *Escherichia coli* AcrB [10]–[18] and *Pseudomonas aeruginosa* MexB [19] multidrug transporters. Their structures suggest that both AcrB and MexB span the entire width of the inner membrane and protrude approximately 70 Å into the periplasm. Along with the models of these two HAE-RND transporters, the crystal structures of the other components of these tripartite complex systems have also been determined. These include the outer membrane channels *E. coli* TolC [20] and *P. aeruginosa* OprM [21], as well as the periplasmic membrane fusion proteins *E. coli* AcrA [22] and *P. aeruginosa* MexA [23]–[25].

Currently, no structural information is available for any protein component of the MtrCDE tripartite complex system. To elucidate the mechanism used by this efflux system for multidrug recognition and extrusion, we here describe the crystal structure of the inner membrane MtrD multidrug efflux pump. The findings reveal a novel structural feature that is not found in other known RND efflux pumps.

## Results and Discussion

### Overall Structure of the *N. gonorrhoeae* MtrD Multidrug Efflux Pump

**Figure 1:**
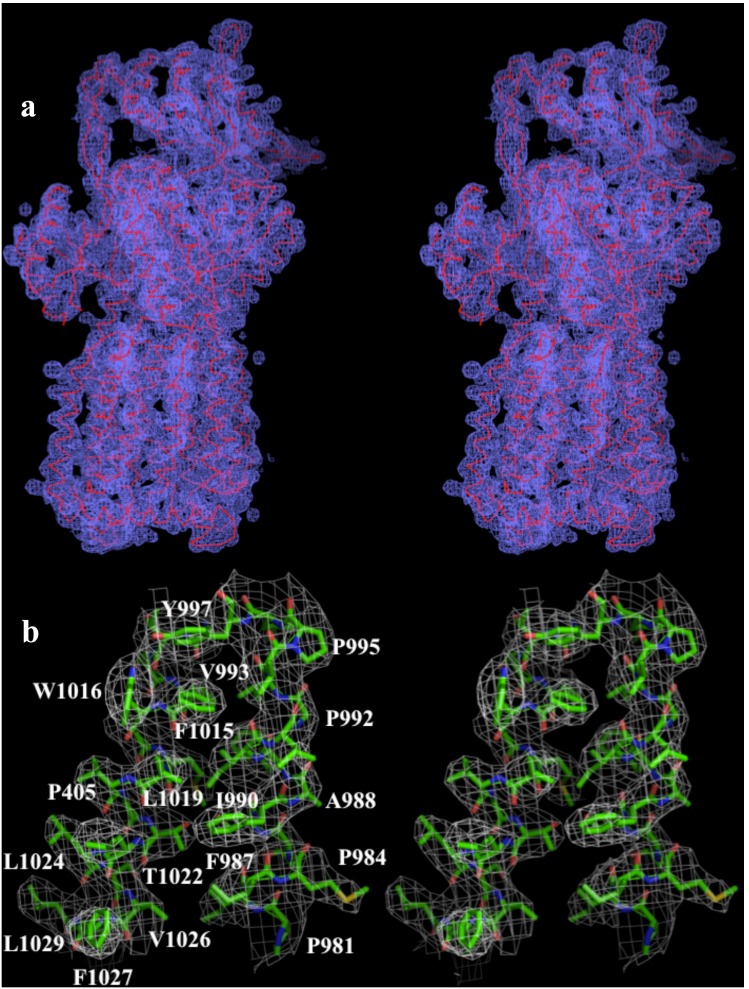
Stereo view of the electron density map of the MtrD efflux pump at a resolution of 3.53 Å. (a) The electron density map contoured at 1.2 σ is in blue. The Cα traces of MtrD are in red. (b) Representative section of the electron density at the interface between TM11 and TM12 of MtrD. The electron density (colored white) is contoured at the 1.2 σ level and superimposed with the final refined model (green, carbon; red, oxygen; blue, nitrogen).

**Figure 2:**
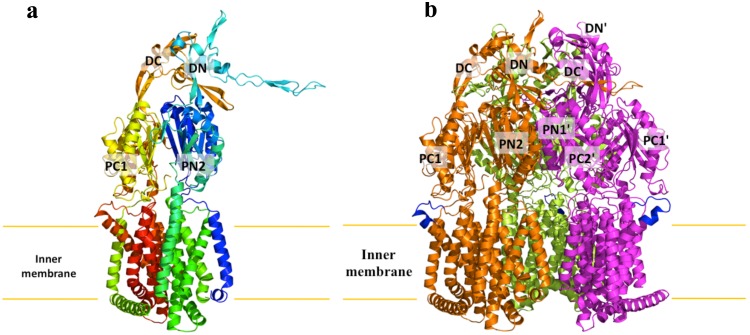
Structure of the *N. gonorrhoeae* MtrD efflux pump. (a) Ribbon diagram of a protomer of MtrD viewed in the membrane plane. The molecule is colored using a rainbow gradient from the N-terminus (blue) to the C-terminus (red). Sub-domains DN, DC, PN2, PC1 and PC2 are labeled. The location of PN1 is behind PN2, PC1 and PC2. (b) Ribbon diagram of the MtrD trimer viewed in the membrane plane. Each subunit of MtrD is labeled with a different color. Residues 917–927 (only found in MtrD) forming the upper portion of TM9 and the loop connecting TM9 and TM10 are in blue color.

We cloned, expressed and purified the full-length MtrD efflux pump containing a 6xHis tag at the C-terminus. We obtained crystals of this membrane protein following an extensive screening for crystallization conditions with different detergents. We then used molecular replacement, utilizing the structure of the “access” protomer of AcrB (pdb code: 2DHH) [12] to determine the three-dimensional structure. The diffraction data can be indexed to the space group *R*32. Data collection and refinement statistics are summarized in Table 1. The resulting electron density maps (Fig. 1) reveal that the asymmetric unit consists of one protomer. The crystal structure of the MtrD multidrug efflux pump has been determined to a resolution of 3.53 Å (Table 1). Currently, 97% of the amino acids (residues 2–493 and 508–1040) are included in the final model (Fig. 2a). The final structure is refined to R_work_ and R_free_ of 27.9% and 33.3%, respectively. The structure of MtrD is closer to the conformation of the “access” protomer of AcrB. However, superimposition of these two structures results in a high RMSD of 7.6 Å over 1,000 C^α^ atoms, suggesting that there are significant differences between these two transporters (Fig. S1).

**Table 1:** Data collection and refinement statistics.

Data set	MtrD
**Data Collection**	
Wavelength (Å)	0.98
Space group	*R*32
Resolution (Å)	50–3.53
	(3.68–3.53)
Cell constants (Å)	
a	152.99
b	152.95
c	360.74
α, β, γ (°)	90, 90, 120
Molecules in ASU	1
Redundancy	2.9 (2.9)
Total reflections	377,955
Unique reflections	20,296
Completeness (%)	97.7 (98.0)
R_sym_ (%)	7.7 (42.9)
I/σ(I)	17.16 (1.9)
**Refinement**	
Resolution (Å)	50–3.53
R_work_	27.9
R_free_	33.3
rms deviation from ideal	
bond lengths (Å)	0.003
bond angles (°)	0.794
**Ramachandran**	
most favoured (%)	89.6
additional allowed (%)	10.1
generously allowed (%)	0.3
disallowed (%)	0

**Figure 3:**
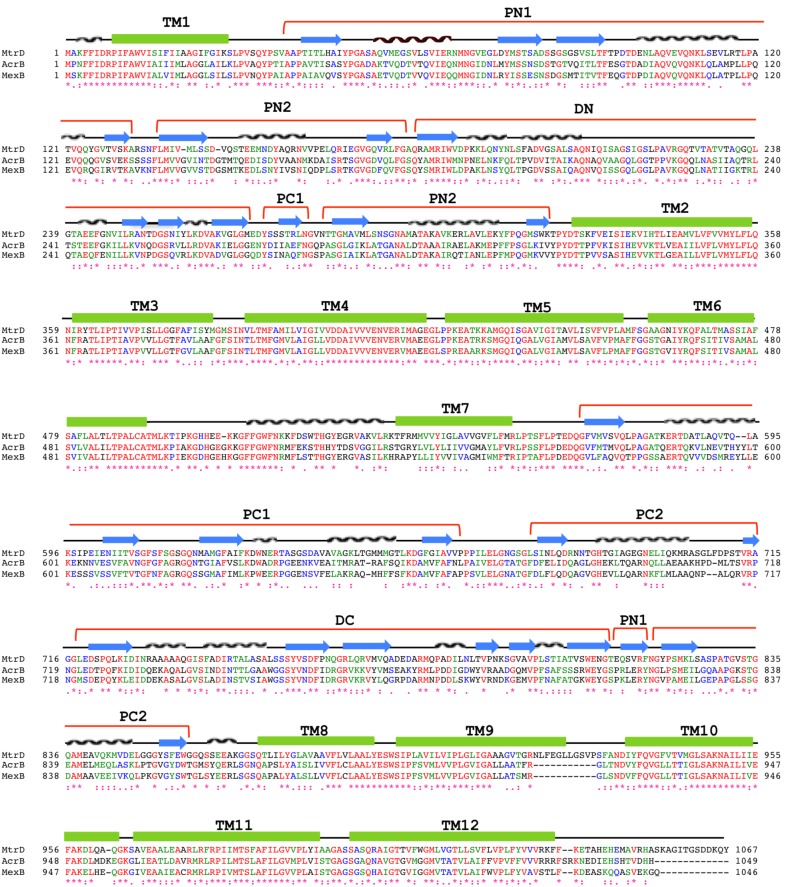
Sequence and topology of MtrD, AcrB and MexB. Alignment of the amino acid sequences of MtrD, AcrB and MexB were done using CLUSTAL W (*, identical residues; :, >60% homologous residues). Secondary structural elements are indicated: TM, transmembrane helix; Nα and Nβ, helix and strand, respectively, in the N-terminal half; Cα and Cβ, helix and strand, respectively, in the C-terminal half. The MtrE docking domain is divided into two sub-domains, DN and DC; whereas the pore domain is divided into four sub-domains, PN1, PN2, PC1 and PC2. The sequence and topology of MtrD are shown at the top.

MtrD assembles as a 125-Å long and 95-Å wide homotrimer (Fig. 2b). Each protomer comprises 12 transmembrane helices (TM1–TM12). Like other RND transporters, the N-terminal (TM1–TM6) and C-terminal (TM7–TM12) halves of MtrD are related by a pseudo-twofold symmetry. A large periplasmic domain is created by two extensive periplasmic loops connecting TM1 with TM2 and TM7 with TM8, respectively. As in AcrB [10]–[18] and MexB [19], this periplasmic domain can be divided into six sub-domains: PN1, PN2, PC1, PC2, DN and DC (Figs. 2 and 3). Sub-domains PN1, PN2, PC1 and PC2 form the pore domain, with PN1 making up the central pore and stabilizing the trimeric organization. However, sub-domains DN and DC contribute to form the docking domain, presumably interacting with the outer membrane channel MtrE. The trimeric MtrD structure suggests that sub-domains PN2, PC1 and PC2 are located at the outermost core of the periplasmic domain, facing the periplasm. Sub-domains PC1 and PC2 also form an external cleft, and this cleft is open in the MtrD structure (Fig. 2). Based on the co-crystal structure of CusBA [26], [27] of the CusCBA tripartite efflux system [28]–[33], the upper regions of PN2, PC1, PC2 and sub-domains DN and DC should directly interact with the MtrC membrane fusion protein to form a functional adaptor-transporter complex.

The *N. gonorrhoeae* MtrCDE tripartite efflux system has the advantage that all these protein components are encoded by the same operon. Thus, the interactions between different proteins are likely to be specific, facilitating analyses of how different components function cooperatively.

### Periplasmic Multidrug Binding Site

Crystallization of AcrB with a variety of substrates [12], has identified that the periplasmic cleft of the pump forms several mini-binding pockets within the extensive, large periplasmic multidrug binding site. This site is supposed to play a predominant role in the selection of drugs for export. Protein sequence alignment reveals that many of the amino acids forming the large periplasmic binding site of AcrB are conserved among MexB and MtrD, indicating that these three multidrug efflux pumps may have a similar substrate binding profile for drug recognition. These conserved amino acids in MtrD include several charged and polar residues, such as S79, S134, R174, D272, E669 and R714, and aromatic residues, such as F136, F176, F610, F612 and F623 (Fig. 3).

A flexible loop is found inside the large periplasmic cleft (Fig. S1), which form the multidrug binding site of the pump. This flexible loop is located deep inside the cleft between subdomains PC1 and PC2, composed of residues 608–619, and should correspond to the Phe-617 loop [16] in AcrB. The loop is highly conserved among MtrD, AcrB and MexB. It is expected that this flexible loop is important for drug recognition and extrusion. There is a chance that this loop may shift positions during the course of the extrusion process to facilitate drug export.

### Transmembrane Helix TM9

**Figure 4:**
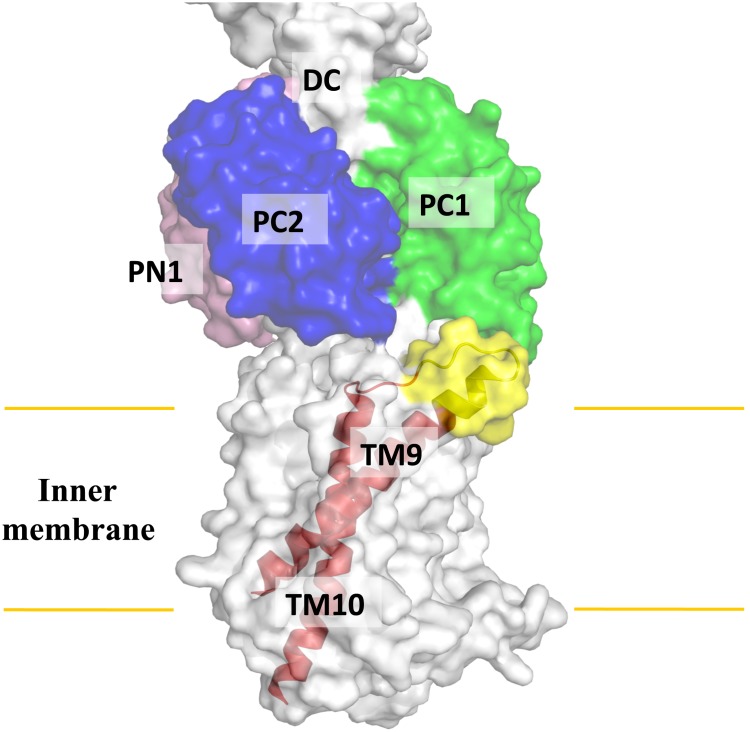
Spatial arrangement between TM9 and the periplasmic cleft. TM9 is inclined from the horizontal membrane plane by 54°. The extra feature (yellow), which is only found in the MtrD pump within the family, is located right next the cleft formed by subdomains PC1 and PC2.

Perhaps the most interesting secondary structural feature appears in TM9 of the MtrD pump. In contrast to other known structures of the RND transporters, MtrD contains an extended region that protrudes into the periplasm and contributes part of the periplasmic domain (Figs. 2 and 3). This region (residues 917–927) comprises an α-helix extending from the upper portion of TM9 and also to the loop connecting TM9 and TM10. Protein sequence alignment suggests that these extra residues are only found in MtrD and not other homologous RND proteins. Therefore, this fragment should represent a unique feature of this pump that cannot be found in other RND pumps. TM9 is distinct in that it is not vertically oriented. Instead, it is inclined from the horizontal membrane plane by 54°. The spatial arrangement between the extra elongated helix and loop (upper portion of TM9) and the periplasmic cleft formed between PC1 and PC2 suggests that these extra structural features may help the pump to transport its substrates more effectively from the outer leaflet of the inner membrane to the multidrug binding site at the periplasmic domain (Fig. 4).

### Proton-relay Network

**Figure 5:**
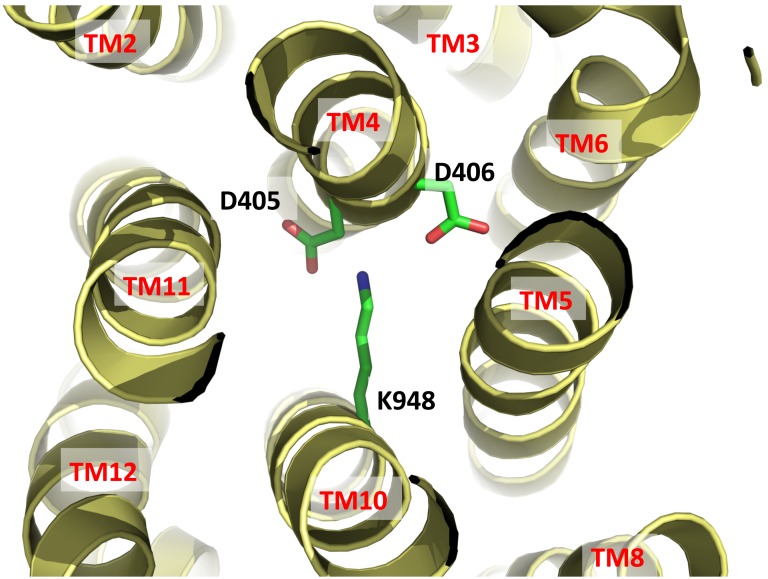
Ion pairs in the transmembrane domain viewed from the cytoplasmic side. Residues D405 and D406 of TM4 and K948 of TM10 that form ion pairs, which may play an important role in proton translocation, are in green sticks.

Drug export by RND transporters is proton motive force (PMF)-dependent. Based on the crystal structure of MtrD, it is expected that the charged residues D405 and D406 of TM4 and K948 of TM10 are important for forming the proton-relay network of the pump (Fig. 5). These residues are supposed to undergo protonation and deprotonation within the transport cycle. The involvement of these charged amino acids in proton translocation was supported by a previous study that showed mutations of these residues inhibits proton translocation [34]. In turn, the MtrE outer membrane channel protein is blocked and unable to dissociate from the MtrCDE tripartite efflux complex [34].

The MtrD multidrug efflux pump should operate through an alternating-access mechanism, similar to the AcrB transporter. Thus, the pump has to go through the transport cycle, which should involve different transient conformations, including the “access”, “binding” and “extrusion” states of this protein. In the MtrD trimer, the conformations of the three protomers are identical to each other, suggesting that these protomers represent the same transient state. In comparison with the structures of AcrB, the conformation of MtrD is closer to that of the “access” protomer of AcrB (Fig. S1). Therefore, our MtrD structure should represent the “access” transient state of the pump. It is expected that this pump will change in conformation to go through the cycle. This conformational change should be coupled to the PMF initiated by the proton-relay network.

## Methods

### Cloning, Expression and Purification of the Inner Membrane MtrD Efflux Pump

Briefly, the full-length MtrD membrane protein containing a 6xHis tag at the C-terminus was overproduced in *E. coli* C43(DE3)*ΔacrB* cells, which harbor a deletion in the chromosomal *acrB* gene, possessing pET15bΩ*mtrD*. Cells were grown in 12 L of 2xYT medium with 100 µg/ml ampicillin at 25°C. When the OD_600_ reached 0.6, the culture was treated with 1 mM IPTG to induce *mtrD* expression, and cells were harvested within 15 h. The collected bacteria were resuspended in low salt buffer containing 100 mM sodium phosphate (pH 7.2), 10% glycerol, 1 mM ethylenediaminetetraacetic acid (EDTA) and 1 mM phenylmethanesulfonyl fluoride (PMSF), and then disrupted with a French pressure cell. The membrane fraction was collected and washed twice with high salt buffer containing 20 mM sodium phosphate (pH 7.2), 2 M KCl, 10% glycerol, 1 mM EDTA and 1 mM PMSF, and once with 20 mM HEPES-NaOH buffer (pH 7.5) containing 1 mM PMSF. The membrane protein was then solubilized in 2% (w/v) 6-cyclohexyl-1-hexyl-β-D-maltoside (Cymal-6). Insoluble material was removed by ultracentrifugation at 100,000×g. The extracted protein was purified with a Ni^2+^-affinity column. The purity of the MtrD protein (>95%) was judged using 10% SDS-PAGE stained with Coomassie Brilliant Blue. The purified protein was then dialyzed and concentrated to 20 mg/ml in a buffer containing 20 mM Na-HEPES (pH 7.5) and 0.05% Cymal-6.

### Crystallization of MtrD

Crystals of the 6xHis MtrD were obtained using sitting-drop vapor diffusion. The MtrD crystals were grown at room temperature in 24-well plates with the following procedures. A 2 µl protein solution containing 20 mg/ml MtrD protein in 20 mM Na-HEPES (pH 7.5) and 0.05% (w/v) Cymal-6 was mixed with a 2 µl of reservoir solution containing 30% PEG 400, 0.1 M Na-Bicine (pH 8.5), 0.1 M NH_4_Cl, 0.05 M BaCl_2_ and 9% glycerol. The resultant mixture was equilibrated against 500 µl of the reservoir solution. Crystals of MtrD grew to a full size in the drops within a week. Typically, the dimensions of the crystals were 0.2 mm×0.2 mm×0.2 mm. The crystals were flash-cooled, using solution containing 40% PEG 400, 0.1 M Na-Bicine (pH 8.5), 0.1 M NH_4_Cl, 0.05 M BaCl_2_, 9% glycerol and 0.05% Cymal-6 as a cryoprotectant before data collection.

### Data Collection, Structural Determination and Refinement

All diffraction data were collected at 100 K at beamline 24ID-C located at the Advanced Photon Source, using an ADSC Quantum 315 CCD-based detector. Diffraction data were processed using DENZO and scaled using SCALEPACK [35].

Crystals of the MtrD efflux pump belong to the space group *R*32 (Table 1) and the best crystal diffracted x-ray to a resolution of 3.53 Å. Analysis of Matthew’s coefficient indicated the presence of one MtrD protomer (113.69 kDa) per asymmetric unit, with a solvent content of 66.7%.

The structure of MtrD was phased using molecular replacement, utilizing the structure of the “access” protomer of AcrB (pdb id: 2DHH) [12] as a search model. After tracing the initial model manually using the program Coot [36], the model was refined against the native data at 3.53 Å-resolution using TLS refinement techniques adopting a single TLS body as implemented in PHENIX [37] leaving 5% of reflections in Free-R set. Iterations of refinement using PHENIX [37] and CNS [38] and model building in Coot [36] lead to the current model, which consists of 1,025 residues with excellent geometrical characteristics (Table 1).

### Accession Code

Atomic coordinates and structure factors have been deposited in the Protein Data Bank with accession code 4MT1.

## Supporting Information

Figure S1 **Comparison of the structures of the MtrD and AcrB efflux pumps.** (a) Ribbon diagram of protomers of MtrD and AcrB viewed in the membrane plane. This is a superimposition of a subunit of MtrD (red) onto an “access” protomer of AcrB (pdb: 2DHH) (green), indicating that the structures of these two efflux pumps are quite distinct. Superimposition of these two structures result in a high RMSDs of 7.6 Å over 1,000 C^α^ atoms. (b) Front view of the periplasmic clefts, formed by subdomains PC1 and PC2, of MtrD and AcrB. The secondary structural elements of these two transporters are colored red (MtrD) and green (AcrB). (c) Potential multidrug binding within the periplasmic cleft of MtrD. The flexible loop created by residues 608–619 is colored blue. This loop should correspond to the Phe-617 loop in AcrB. It is suspected that this flexible loop may swing into this drug binding site to facilitate export during drug extrusion. The rest of the secondary structural elements of MtrD are colored red.
(TIF)
