# Supplementary figures and images for: Crystal Structure of the Neisseria gonorrhoeae MtrD Inner Membrane Multidrug Efflux Pump
Source: PLoS One. 2014 Jun 5;9(6):e97903. doi: 10.1371/journal.pone.0097903 (PMC4046932; doi:10.1371/journal.pone.0097903)

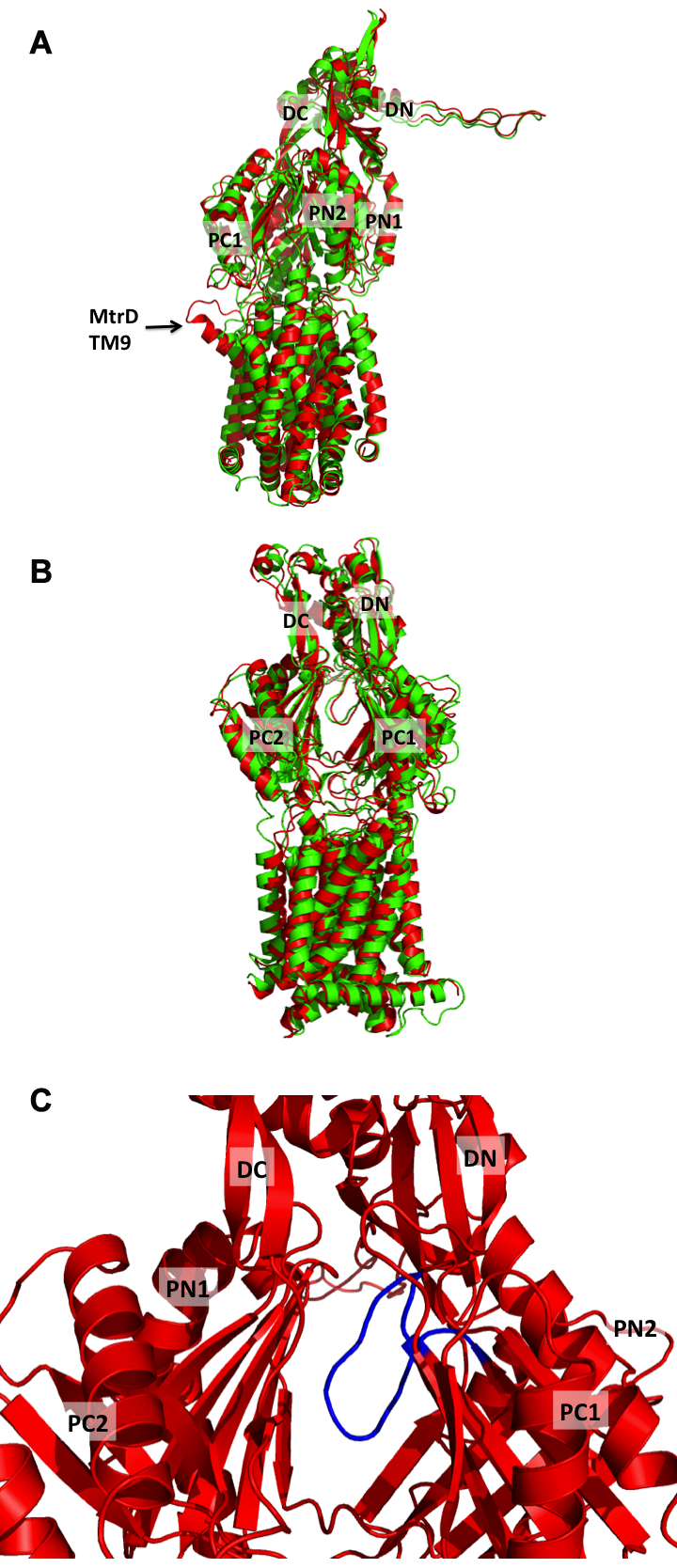

Supplement: Figure S1 — Comparison of the structures of the MtrD and AcrB efflux pumps. (a) Ribbon diagram of protomers of MtrD and AcrB viewed in the membrane plane. This is a superimposition of a subunit of MtrD (red) onto an “access” protomer of AcrB (pdb: 2DHH) (green), indicating that the structures of these two efflux pumps are quite distinct. Superimposition of these two structures result in a high RMSDs of 7.6 Å over 1,000 Cα atoms. (b) Front view of the periplasmic clefts, formed by subdomains PC1 and PC2, of MtrD and AcrB. The secondary structural elements of these two transporters are colored red (MtrD) and green (AcrB). (c) Potential multidrug binding within the periplasmic cleft of MtrD. The flexible loop created by residues 608–619 is colored blue. This loop should correspond to the Phe-617 loop in AcrB. It is suspected that this flexible loop may swing into this drug binding site to facilitate export during drug extrusion. The rest of the secondary structural elements of MtrD are colored red. (TIF) [file pone.0097903.s001.tif]
